# Cost-Benefit of Stockpiling Drugs for Influenza Pandemic

**DOI:** 10.3201/eid1108.041156

**Published:** 2005-08

**Authors:** Ran D. Balicer, Michael Huerta, Nadav Davidovitch, Itamar Grotto

**Affiliations:** * Ministry of Health, Jerusalem, Israel;; † Ben-Gurion University of the Negev, Be'er-Sheva, Israel

**Keywords:** antiviral agents, disease outbreaks, Influenza, Prevention and Control, Cost-Benefit Analysis

## Abstract

We analyzed strategies for the use of stockpiled antiviral drugs in the context of a future influenza pandemic and estimated cost-benefit ratios. Current stockpiling of oseltamivir appears to be cost-saving to the economy under several treatment strategies, including therapeutic treatment of patients and postexposure prophylactic treatment of patients' close contacts.

The widespread epidemic of highly pathogenic avian influenza that emerged in east Asia continues today. As the epidemic grows, so does the probability that this virulent virus will acquire genetic traits for increased person-to-person transmissibility, potentially setting the stage for the next global influenza pandemic (1).

The next pandemic will be associated with major adverse health and economic outcomes, with estimated costs reaching US$166 billion in the United States alone (2). The World Health Organization recently encouraged health authorities to consider stockpiling antiviral drugs in anticipation of a pandemic (3). However, the cost-benefit of stockpiling has yet to be assessed, and the optimal strategy for antiviral use is still under debate. The Israeli Ministry of Health appointed a working group to address national preparation for an influenza pandemic. We set out to identify strategies for the use of the antiviral drug oseltamivir in the containment of a pandemic and to construct a mathematical model to appraise the cost and benefit of each strategy in terms of health-related and economic outcomes.

## The Study

We estimated the health-related impact of pandemic influenza on the Israeli population, by using rates (illness, physician visits, hospitalizations, and deaths) derived from previous pandemics, according to Meltzer et al. (2). Costs related to these outcomes were calculated from data provided by a major Israeli healthcare organization (4) and by the Israeli Central Bureau of Statistics (5). We calculated direct costs to the healthcare system and overall costs to the economy, the latter including the value of lost workdays but not the potential value of lost lives. Point estimates of variables used in the base-case model are detailed in Table 1 and Appendix.

**Table 1:** Point estimate and range of values for selected model variables*

Variable	Point estimate	Range
Overall attack rate, %†	25	15–35
Probability of pandemic (per year)	3	1–10
Adult workdays lost, by age, y		
<1–≤18	3.7	2–5
19–64	4.9	3–7
≥65	0.5	0.25–2
Average hospital stay (days) by age, y		
<1–≤18	4.0	2–5
19–64	5.8	2–7
≥65	7.0	4-9
Patients seeking medical care within 48 h, %	80	70–90
Efficacy of antiviral prophylaxis, %		
Preexposure prophylaxis (50 days)	71	57–85
Postexposure prophylaxis (7 days)	36	25–47
Efficacy of antiviral therapy, %		
Reduction in hospitalizations	59	30–70
Reduction in antimicrobial drug use	63	40–80
Reduction in lost workdays under treatment	1	0.5–1.5

*Complete list and references available in online Appendix. †Population attack rate was calculated by stratifying the population by age and risk and applying age- and risk-specific attack rates and ranges (Appendix).

According to base-case assumptions, a pandemic would result in an estimated 1,618,200 patients (≈25% of the Israeli population), 781,921 physician visits, 10,334 hospitalizations, 2,855 deaths, and 6,536,240 lost workdays. These outcomes would result in an excess of $55.4 million in health-related costs and in overall costs to the economy of $523.5 million (≈0.5% of the Israeli gross domestic product).

We defined 3 strategies for the use of antiviral drugs during a pandemic: therapeutic use, long-term preexposure prophylaxis, and short-term postexposure prophylaxis for close contacts of influenza patients (with index patients under treatment). The first 2 strategies could target either the entire population or only those at high risk for complications. The efficacy of therapeutic treatment was based on currently available evidence regarding epidemic influenza (Appendix). Systematic review and meta-analysis were used to estimate the efficacy of preexposure prophylaxis, while the expected efficacy of postexposure prophylaxis and the number of persons treated under this strategy were estimated by using the results of a recently published stochastic simulation model (6).

**Figure A1:**
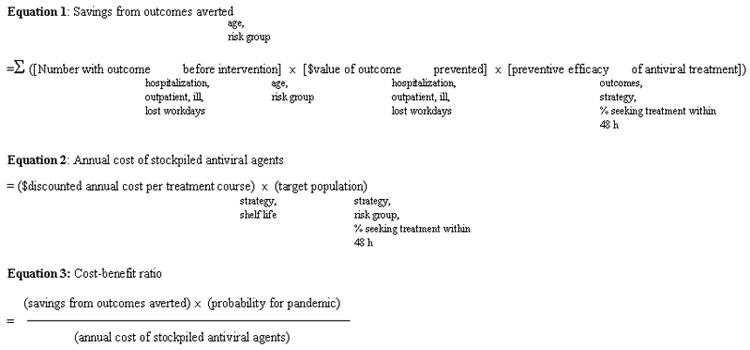
Formulas used to analyze the impact of each strategy on health-related outcomes.

The impact of each strategy on health-related outcomes was analyzed in a spreadsheet model by using the formulas summarized in Figure A1. Briefly, the economic benefit of each strategy was calculated by multiplying each of the reductions in adverse outcomes by its estimated economic value. The cost of each strategy was calculated by multiplying the estimated number of treated persons by the discounted cost of a single antiviral course. Oseltamivir was selected as the drug of choice, at a daily dosage of 75 mg for prophylaxis and 150 mg for treatment (7). Oseltamivir stockpiling costs were calculated with prices quoted in March 2004 by the manufacturer's representative in Israel for uncapsulated, water-soluble, bulk active powder with a 10-year shelf life.

We compared the economic outcomes of each of the 5 strategies with nonintervention, estimated stockpiling costs, and calculated cost-benefit ratios. Based on the historic incidence of 3 influenza pandemics over the last century, we adjusted all cost-benefit outcomes for a conservatively estimated probability of 3 pandemics every 100 years and applied a wide range of estimates for sensitivity analyses (Appendix). Table 2 details the cost-benefit ratios of the competing strategies. Therapeutic treatment and postexposure prophylaxis were shown to be cost-saving, with a cost-benefit ratio of 2.44–3.68.

**Table 2:** Cost-benefit ratios of antiviral utilization strategies*

Strategy		Cost-benefit ratio, relative to nonintervention	
		All costs to economy	Direct healthcare costs
NI	No intervention (base case)	Ref.	Ref.
1a	Therapeutic use (all patients)	2.44	0.30
1b	Therapeutic use (limited to patients at high risk)	3.68	1.51
2a	Preexposure long-term prophylaxis of entire population	0.38	0.04
2b	Preexposure long-term prophylaxis, limited to high-risk population	0.37	0.10
3a	Postexposure short-term prophylaxis for all close contacts ("ring prophylaxis"), including treatment of index patients	2.49	0.27

*Ref., reference value of zero divided by zero.

Since many characteristics of the next pandemic viral strain remain unknown, our modeling methods and parameter estimates were designed to consistently underestimate intervention-related benefits, thus yielding minimum estimates of the true cost-benefit ratios. In a series of multivariate sensitivity analyses that used the variable ranges detailed in Table 1, the model proved to be robust. Even under the most unfavorable estimates, prepandemic stockpiling remained cost-saving as long as the estimated probability of a pandemic remained >1 every 80 years. No consistent advantage to either therapeutic or short-term prophylactic use of antiviral drugs could be determined.

## Conclusions

In light of recent episodes of human infection with avian influenza, the World Health Organization reiterated its 1997 call for all countries to prepare for the next "inevitable, and possibly imminent" pandemic (3). Strain-specific vaccine, the most effective tool for influenza control, will most likely not be available in the early stages of a pandemic because of its prolonged development time (3), and early control measures will have to employ alternative options, mainly the judicious use of antiviral drugs. These drugs are likely to be in short supply if not preemptively stockpiled (3). Compared with neuraminidase inhibitors, the M2-inhibitor drugs have several major disadvantages, mainly high rates of viral resistance (as shown in recent H5N1 and H9N2 isolates) and adverse effects (8).

Our model suggests that prepandemic stockpiling of oseltamivir is cost-saving to the economy over a wide range of treatment strategies. Stockpiling is also directly cost-saving to the healthcare system, if oseltamivir use is limited to treating patients at high risk. Investment in stockpiling remains cost-saving to the economy as long as the estimated annual pandemic risk remains >1 pandemic every 80 years. In the last 400 years, at least 31 pandemics have been recorded (8), so that regardless of recent events in Southeast Asia, present investments in antiviral agents can be expected to yield a substantial economic return of >$3.68 per $1 invested, while saving many lives.

This favorable cost-benefit ratio can be achieved if stockpiled antiviral drugs are administered either solely as a therapeutic measure or as short-term prophylaxis for exposed contacts, a strategy termed "ring prophylaxis" (9) or "targeted prophylaxis" (6). Only 1 study published to date (6) used dynamic mathematical modeling to examine the expected effectiveness of this latter control measure on the population level; that study suggested that this strategy may significantly reduce illness and death. This epidemiologically directed short-term prophylaxis of close contacts may require antiviral stockpiles considerably larger than necessary for therapeutically treating patients, but our model suggests that this investment may still prove cost-saving, providing that the outbreak dissemination patterns and population attributes correlate with those assumed by Longini et al. (Appendix).

When one considers a ring prophylaxis strategy, the risk of "strategy failure" due to early antiviral stockpile depletion must be considered. If postexposure prophylaxis does not confer sufficient immunity upon exposed contacts who underwent prophylaxis, and if vaccines or additional antiviral agents do not become available, rapid consumption of available stocks may leave the population vulnerable to additional outbreak waves, potentially caused by influx of new cases. The probabilities of similar "failure" scenarios are difficult to assess and were not included in our analysis. Application of this strategy for the entire population without surplus antiviral reserves should therefore be considered cautiously and monitored closely.

This study aimed to elicit minimum cost-benefit estimates for investment in a national antiviral stockpile. Among our conservative assumptions, we chose to exclude indirect costs of preventable deaths, which, if added, would have increased cost-benefit ratios up to 6-fold (Appendix). Furthermore, in view of recent events in east Asia, the probability of a pandemic has probably risen to >3 per 100 years, and new strains may prove more pathogenic than previous pandemic strains. In modeling the benefits of therapeutic strategies, we omitted the beneficial effects of decreased viral shedding afforded by neuraminidase inhibitors (7), such as a lower secondary attack rates among untreated contacts. We also ignored the possibility that a fully implemented prophylactic strategy might achieve full containment of the outbreak (probability estimated at ≈6% for 7-day postexposure ring prophylaxis (6), dependent on several factors such as compliance, delay in treatment initiation, and basic reproductive number (6,10). Finally, as witnessed during the epidemic of severe acute respiratory syndrome (SARS), the economic consequences of a rapidly disseminating disease extend well beyond direct costs to the healthcare system and lost workdays. Canada had losses >$1 billion during the SARS epidemic, although the disease directly affected <500 patients (11). From an economic viewpoint, mitigating a pandemic could prevent extensive indirect economic losses.

The conclusions of this study must be considered carefully during the planning of antiviral stockpiling. Drug prices can be expected to change substantially as a result of contractual negotiations with manufacturers (although our results indicate stockpiling may remain cost-saving even if drug costs are more than tripled, as would be the case if preprepared capsules are purchased). Powder-form antiviral drugs have considerable advantages in terms of cost and shelf life, but the logistical aspects of their preparation and distribution should be further assessed to confirm feasibility. Finally, we assumed that strain-specific vaccine would not be available in sufficient quantities during the first stages of the pandemic. Efforts are currently being directed towards shortening this delay. Once available, strain-specific vaccines would likely be the favored intervention, with antiviral agents serving as adjunct treatment.

In summary, prepandemic stockpiling of antiviral drugs can be expected to prove cost-saving. Cost-beneficial strategies for their use may involve treatment of patients, and, if backed by adequate antiviral stockpiles, short-term postexposure prophylaxis of close contacts. These strategies should be considered when planning stockpiling efforts.

Several countries have already begun active stockpiling efforts (12), sufficient in some cases to allow antiviral treatment of up to 25% of the population (13). We believe that antiviral stockpiling should be considered a prudent investment that may help mitigate this impending global threat.

## Appendix

### Cost-Benefit of Stockpiling Antiviral Agents for Influenza Pandemic

#### Detailed Methods, Data, and Results

We used a static spreadsheet model (Microsoft Excel 2000, Microsoft Corporation, Redmond, WA, USA) to estimate the effect of pandemic influenza on the Israeli population. We defined 3 separate strategies for the use of antiviral drugs during a pandemic, analyzed the effect of these strategies on pandemic outcomes, and estimated the economic consequences of each scenario.

#### Health-related Outcomes of the Pandemic

We divided the population (6,748,000 in Israel at the end of 2003) into 3 age categories: ≤18 years, 19–64 years, and ≥65 years (14). Each age category was further grouped by low- or high-risk for serious complications of influenza infection by using the US population age-specific proportions (15). When we adjusted for the age structure of the Israeli population, the high-risk group numbered ≈899,000 persons (14% of total population). Point estimates and ranges of health-related variables used in the base-case model are detailed in Table A1.

We constructed a baseline nonintervention scenario by using age- and risk-specific rates to estimate the expected numbers of patients, physician visits, hospitalizations, deaths, and lost work days (15) and calculated the economic value of each of these outcomes. These outcomes were then recalculated for each intervention strategy, as described in detail below, yielding the economic benefit associated with each strategy. The resulting figure was then compared to the costs associated with purchasing enough antiviral stocks to implement the strategies.

Economic Outcomes

Point estimates of economic variables used in the base-case model are detailed in Table A2. Cost of patient visits (including cost of prescription drugs and diagnostic tests) was based on current data provided by a major healthcare organization in Israel (16). Hospitalization and workday costs were provided by the Israeli Central Bureau of Statistics (14). The benefit of each strategy was defined as the cost savings it yielded, relative to nonintervention. We calculated both direct costs to the healthcare system and overall costs to the economy, the latter including the value of lost workdays but not indirect costs from excess deaths.

According to previous assessments by Meltzer et al. that addressed a similar scenario (15), the value of lives lost made up 83% of all estimated costs of pandemic influenza. When applying these estimates to our study, the addition of the value of lives lost increases our calculated cost-benefit ratios >6-fold.

#### Drug Selection and Costs

Drug resistance may appear in approximately one third of patients treated with the M2 inhibitors amantadine or rimantadine (17,18), and early rimantadine resistance has been described when this drug was used for treatment and postexposure prophylaxis in families (18). In vitro susceptibility testing of recent human H5N1 isolates from east Asia indicate that this strain is resistant to the antiviral drugs amantadine and rimantadine but susceptible to the neuraminidase inhibitor oseltamivir (19). Furthermore, oseltamivir has been shown to be effective against H5N1 and H9N2 viruses in mice (20). M2 inhibitors have several other major disadvantages, including higher rates of adverse effects and no proven efficacy for reducing influenza complication rates. Of the 2 currently available neuraminidase inhibitors, oseltamivir and zanamivir, only oseltamivir is licensed for prophylaxis in Israel (21), the United Kingdom (22), and the United States (23). We therefore limited our analysis to oseltamivir, at a daily dose of 75 mg when used for prophylaxis (24) and 150 mg when used for treatment (25). Per diem oseltamivir costs were calculated for each strategy by using the projected number of neuraminidase inhibitor recipients and drug prices quoted in March 2004 by the manufacturer's representative in Israel (Roche Pharmaceuticals [Israel] Ltd, pers. comm.). Parameters related to drug costs are detailed in Table A2. We based our calculations on a 10-year shelf life for the drug, when stored as bulk active powder, and discounting was performed by using the locally accepted annual rate of 3%. The cost-benefit ratio of each strategy was calculated by dividing strategy-specific drug costs by treatment-derived economic benefits. Ratios were calculated separately for direct health-related costs and for overall costs to the economy. Since the different strategies were not mutually exclusive, incremental cost-benefit analysis was not performed.

#### Probability of a Pandemic

We adjusted all cost-benefit outcomes for the estimated probability of a pandemic occurring per year. We based our calculations on the recent historic incidence of 3 influenza pandemics over the last century, thus adopting a conservative point estimate of 1 pandemic every 33 years for the base-case and applied a wide range of estimates for sensitivity analyses.

#### Nonintervention

The baseline scenario, modeled by using estimates derived by Meltzer et al. from previous pandemics (15) and data collected in Israel during interpandemic periods (16), provides an estimate of health-related and economic outcomes that would be expected were the pandemic allowed to run its natural course. These estimates by Meltzer et al. are based on a wide range of possible attack rates (15%–35%). Illness and death rates in these estimates are considerably lower than those estimated for the pathogenic potential of the currently circulating H5N1 avian strain. These estimates were selected to be in line with our general approach to underestimate the pandemic's effect and potential benefits provided by each of the interventions, as explained below. The parameters used for these calculations are detailed in Tables A1 and A2, and the formulas are detailed in Figure A1. This scenario serves as a reference category against which the alternative strategies can be compared.

#### Intervention Strategies

We assumed that strain-specific vaccine would be unavailable during the initial months of a pandemic. If appropriately stockpiled, antiviral drugs can be directed either at therapy or prophylaxis. Prophylactic strategies can be divided into long-term preexposure prophylaxis and short-term, epidemiologically directed postexposure prophylaxis. We compared each of 3 strategies with nonintervention, alternately targeting either the entire population or only populations at high risk for complications.

##### Strategy 1: Therapeutic Antiviral Use

When given therapeutically to influenza patients within 48 hours of symptom onset and if continued for 5 days, neuraminidase inhibitors can reduce the duration of clinical symptoms by an average of 1 day (26), hospitalization rates by 59% (27), and antimicrobial drug use by 63% (26) (Table A3). Since the effects of treatment on reducing death rate have not yet been studied or quantified, we have not included in our model this or other yet-unproven potential benefits of these drugs, in keeping with our approach toward underestimating any benefits of intervention. Moreover, effects on death rate reduction would not alter our cost-benefit calculations, since only illness-related costs were taken into account.

We evaluated 2 variations of therapeutic antiviral use: nonselective treatment available to all patients (strategy 1A) and selective treatment limited to use in patients at high risk (strategy 1B). We adjusted this model for treatment initiation rates, since not all patients would be expected to reach a physician and initiate treatment within the 48-hour window of therapeutic opportunity. The proportion of patients likely to seek physician care was estimated by using data available from studies of interpandemic influenza in Israel (16), and the wide range of estimates was used in the sensitivity analysis to address treatment-seeking behavior during a crisis.

##### Strategy 2: Preexposure Prophylaxis

We evaluated 2 variations of preexposure antiviral use: mass prophylaxis made available to the entire population (strategy 2A) and selective prophylaxis, limited to groups at increased risk for complications (strategy 2B). We assumed a pandemic duration of 50 days (30) and calculated drug costs accordingly.

We performed a systematic review and meta-analysis of studies evaluating the protective efficacy of neuraminidase inhibitors when used for preexposure (seasonal) prophylaxis. The methods and results of this meta-analysis are described below.

A computerized search was conducted by using MEDLINE (January 1966–December 2004) and Embase (January 1980–December 2004) databases. The following combination of keywords was used: (influenza) and ([oseltamivir or Tamiflu] or [zanamivir or Relenza]) and (prevention or prophylaxis or chemoprophylaxis). This search was limited to articles published in English. In addition, we searched these databases using the names of authors of studies identified in the primary search and in the studies' reference sections. We also contacted drug companies for information on unpublished trials.

Our search identified 24 candidate papers. These papers were then independently reviewed by 2 of the authors (R.D.B. and I.G.), 1 of whom was blinded to authors' names, journal, date of publication, and site of study. Of the candidate papers, we selected randomized, controlled, double-blind trials that met all of the following criteria: 1) evaluated preexposure (seasonal) prevention of naturally occurring influenza with zanamivir or oseltamivir (oseltamivir 75–150 mg/day, zanamivir 10 mg/day); 2) included a study sample whose participants were 18–69 years of age; 3) included a healthy, community-based population without specific baseline disease; 4) provided data on intent-to-treat analysis; and 5) reported the incidence of laboratory-confirmed influenza in placebo and control groups. Two studies met these inclusion criteria (24,28). Table A4 summarizes the main features of the studies included in the analysis.

The 2 authors who reviewed the papers also abstracted information from each of the selected studies. In cases in which >1 article was published on the same study, all articles were assessed for data consistency. All data were abstracted by using a standardized protocol and computerized report form. Reported relative risk (RR), or incidence data necessary for computing RR, were abstracted based on intent-to-treat analysis.

For all studies included in the analysis, cases were defined as clinical influenza confirmed by isolation of influenza virus, by reverse-transcriptase polymerase chain reaction (RT-PCR), or by testing paired serum samples for rise in antibody titer against circulating influenza virus. For each study, we calculated the RR and 95% confidence interval (CI) for influenza infection in the intervention group compared to the control group. We calculated the overall RR for preexposure prophylaxis. Since oseltamivir and zanamivir are both neuraminidase inhibitors and the results of the studies that evaluated these drugs were comparable, we combined the results of these studies in our overall estimate.

In each study, 95% CIs of the RR were calculated by using Breslow's method (31). Since the results of the individual studies were homogenous, the overall RRs and 95% CIs were calculated by using precision-based estimates, as described by Fleiss (32) and Kleinbaum et al. (33), which assume a homogeneity of effect between studies (fixed-effects model). The protective efficacy of the interventions was calculated as 1 – RR.

Heterogeneity of RRs across n studies was tested with the formula

χ^2^ heterogeneity = ΣW_i_M_i_^2^ – (ΣW_i_M_i_)^2^ / ΣW_i_

where M_i_ is an individual measure of association and W_i_ is a weighting factor equal to the reciprocal of the squared individual variance. Significance was evaluated with n – 1 degrees of freedom.

Since only 2 studies of preexposure prophylaxis were included, no sensitivity analysis was applied. All computations were performed by using PEPI software for epidemiologic analysis (34).

The results of each study and overall estimate of preexposure studies are presented in Table A5. The overall protective efficacy of this intervention was 71% (95% CI 57%–80%). This result was used as the point estimate for the protective efficacy of neuraminidase inhibitors when used in a setting of preexposure prophylaxis (Table A3).

##### Strategy 3: Postexposure Prophylaxis

In this scenario, stockpiled antiviral agents are administered as short-term prophylaxis to exposed close contacts in addition to treating the index patients, a strategy termed "ring prophylaxis" (35) or "targeted prophylaxis" (29) (strategy 3A). Only 1 study (29) published to date used dynamic mathematical modeling to examine the expected effectiveness of this pandemic control measure on a population level during a pandemic. This stochastic simulation model by Longini et al. suggested that during an influenza pandemic, postexposure prophylaxis targeted at close contacts (i.e., household, daycare centers, play groups, and schools) might prevent 36% of all infections while providing prophylactic antiviral courses to 54.8% of the population. We have adopted these results as point estimates, while allowing a wide range of values for our sensitivity analysis (Table A3).

While these estimates are the only ones published to date on the efficacy of this strategy during an influenza pandemic, the inherent limitations of stochastic models such as the one used by Longini et al. must be acknowledged when this strategy is considered in practice. The authors of that study modeled communities of 2000 people, with predefined mixing patterns between subpopulations and a relatively limited number of "outsiders" entering the population. The paucity of evidence-based data on contact and transmission probabilities regarding both epidemic and pandemic influenza, as well as the complexity of real-life mixing patterns within a large population, may lead to substantial alterations in the efficacy of targeted prophylaxis, which cannot be modeled. Longini et al. have shown that these estimates are sensitive to various (currently unpredictable) parameters of the population and the pandemic strain, such as population compliance, delay in treatment initiation, and the outbreak basic reproductive number (29,36). In addition, as this strategy requires relatively rapid use of large antiviral stocks, it may potentially lead to premature depletion of the stockpiles. This postexposure prophylaxis strategy is less likely to lead to such early depletion of antiviral stocks, when used in the first stages of the pandemic, as long as relatively few suspected cases are identified. In these first stages, longer prophylactic courses may be considered. As the scale of the outbreak increases, application of this strategy becomes more problematic, as it may lead to rapid consumption of stockpiled drugs, and should therefore be monitored closely.

##### Breakthrough Cases

Under strategies 2 and 3, clinical influenza would develop in a varying proportion of participants who received prophylaxis despite treatment (breakthrough cases). We assumed that these patients, although becoming ill, would nonetheless benefit from the neuraminidase inhibitors that they had been receiving, and they were credited with the effects of therapeutic neuraminidase inhibitor treatment, such as shorter duration of illness and fewer hospitalizations and deaths (see strategy 1).

#### Sensitivity Analyses

Very little evidence-based data are currently available to allow accurate predictions regarding the effect of the next influenza pandemic. This model, as well as similar published models that attempted to make inferences regarding an impending pandemic, is based on estimates derived mainly from sparse data on previous pandemics and on the characteristics of interpandemic influenza. We have systematically chosen the most conservative estimates available in the literature regarding the different parameters, but such estimates are still associated with much uncertainty. A series of sensitivity analyses was therefore conducted to establish the robustness of the various outcomes of this model. These analyses applied a wide range of values for the parameters relating to pandemic probability, health-related pandemic outcomes, and antiviral drug efficacy. We considered the economic parameters as relatively stable, as they are based on verifiable data. Tables A1 and A3 list the variables used for strategy outcome estimates and the ranges used for sensitivity analyses.

The sensitivity analysis examined the effect that the modification of each parameter had on the main outcomes (cost-benefit ratios), thus assessing to which of the variables the model was most sensitive. This analysis showed that the most important parameters were the severity of the pandemic (illness and death rates) and the annual probability of a pandemic. Reducing the annual probability of a pandemic to 1 every 100 years, only the strategy of treating patients at high risk represented a cost savings, with a cost-benefit ratio of 1.23. When a combination of low-range estimates (according to Meltzer et al.) of the various health-related pandemic outcomes modeled were assumed, the cost-benefit ratios of therapeutic treatment and postexposure prophylaxis were decreased, but these strategies remained cost-saving, with cost-benefit ratios of >2.27 and 1.47, respectively. We then analyzed our data to define the ranges of cost-benefit ratios when applying various combinations of the parameters in Table A1. The results of these analyses are presented in Table A6. Only when we assumed an annual pandemic probability of 1 per 100 years, together with the most unfavorable sets of estimates for the other parameters, did all of the intervention strategies become non–cost-saving. However, if the annual probability of a pandemic remains >1 every 80 years, stockpiling antiviral drugs to treat patients at high risk remains consistently cost-saving, even when one assumes that the most unfavorable sets of estimates within the ranges applied for all other parameters. Since current events in Southeast Asia suggest that the probability of a pandemic may now be 2–3 times greater than our upper limit (1 every 10 years), cost-benefit ratios can be adjusted by multiplying current high-end estimates by the same factor. Additionally, health-related effects of an H5N1 pandemic may be similar or worse than those seen in 1918, with higher attack rates, complication rates, hospitalizations, and death rates. If, for example, under our assumed high-end attack rates, the rates of physician visits and hospitalizations are double our upper value, the cost-benefit ratios of therapeutic treatment of all patients and postexposure prophylaxis would be 6.55 and 3.12, respectively. We did not pursue higher ranges for these values, as our basic conclusion regarding the cost-benefit of currently purchasing antiviral drugs does not change.

## Appendix

**Table A1:** Health-related parameters included in the model

Variable by age	Low risk		High risk		
	Point estimate	Range	Point estimate	Range	Reference
Attack rates (%)					
Overall	25	15–35	25	15–35	15
≤18	38.50	23–54	38.50	23–54	15
19–64	17.50	11–25	17.50	11–25	15
≥65	15.50	9–22	15.50	9–22	15
Outpatient visits (per person)					
≤18	0.1975	0.165–0.23	0.346	0.259–0.403	15
19–64	0.0625	0.04–0.085	0.1095	0.07–0.149	15
≥65	0.0595	0.045–0.074	0.1045	0.079–0.13	15
Hospitalizations (per 1,000)					
≤18	0.5	0.2–2.9	2.9	2.1–9.0	15
19–64	1.465	0.18–2.75	2.985	0.83–5.14	15
≥65	2.25	1.5–3.0	8.5	4.0–13.0	15
Deaths (per 1,000)					
≤18	0.024	0.014–0.125	0.22	0.126–7.65	15
19–64	0.037	0.025–0.09	2.91	0.1–5.73	15
≥65	0.42	0.28–0.54	4.195	2.76–5.63	15
Adult workdays lost					
≤18	3.7	2–5	3.7	2–5	16
19–64	4.9	3–7	4.9	3–7	16
≥65	0.5	0.25–2	0.5	0.25–2	16
Average hospital stay (d)					
≤18	4.0	2–5	4.0	2–5	15
19–64	5.8	2–7	5.8	2–7	15
≥65	7.0	4–9	7.0	4–9	15

**Table A2:** Cost and benefit parameters included in the model

Variable	Point estimate (US$)	Reference
Average daily wage*	71.6	14
Cost of hospital day*	317.8	14
Cost of physician visit*†	45.3	17
Antiviral (NAI) drug costs‡		
5-day therapeutic course	8.9	Roche, pers. comm.
7-day prophylactic course	6.2	Roche, pers. comm

*Converted from original prices in NIS (new Israeli shekels) at the rate of US$1 = 4.50 NIS. †Physician costs include prescription drugs and diagnostic tests. ‡NAI, neuraminidase inhibitors. Based on manufacturer's quoted price for oseltamivir.

**Table A3:** Neuraminidase efficacy and related parameters included in the model

Variable	Point estimate	Range	Reference
Efficacy of antiviral prophylaxis*			
Preexposure prophylaxis†	71	57–85	24,28
Postexposure prophylaxis†	36	25–47	29
Efficacy of antiviral therapy			
% reduction in hospitalization	59	30–70	27
% reduction in antimicrobial drug use	63	40–80	26
Reduction in lost work days under treatment	1	0.5–1.5	26
Annual probability of pandemic (%)	3	1–10	Assumed
% patients seeking medical care within 48 hours	80	70–90	16

*Efficacy presented as percent reduction of attack rate. †Based on a meta-analysis of 2 studies (see text for details).

**Table A4:** Preexposure (seasonal) prophylaxis trials included in the analysis

Trial	Age group, y	Intervention (no. participants)	Treatment duration
Hayden et al., 1999 (11)	18–65	Oseltamivir 75 mg 1×/d (520)	6 weeks
		Oseltamivir 75 mg 2×/d (520)	
		Placebo (519)	
Monto et al., 1999 (15)	18–69	Zanamivir 10 mg 1×/d (553)	4 weeks
		Placebo (554)	

**Table A5:** Relative risk (RR) and 95% confidence interval (95% CI) for influenza infection and protective efficacy of individual trials with estimate of preexposure (seasonal) prophylaxis

Trial	Drug	RR (95% CI)	Protective efficacy (%)	p value for heterogeneity
Hayden et al. (11)	Oseltamivir	0.26 (0.13–0.50)	74	
Monto et al. (15)	Zanamivir	0.32 (0.17–0.63)	68	
Overall		0.29 (0.20–0.43)	71	0.643

**Table A6:** Cost-benefit ratios when applying combinations of model parameters in a sensitivity analysis

Probability of pandemic	Estimates of health-related pandemic outcomes*								
	Minimum			Base case			Maximum		
	1/100 y	1/33 y	1/10 y	1/100 y	1/33 y	1/10 y	1/100 y	1/33 y	1/10 y
Strategy									
Therapeutic use									
All patients	0.71–0.79	2.13–2.37	7.10–7.92	0.73–0.87	2.18–2.62	7.25–8.74	0.74–0.96	2.23–2.88	7.42–9.61
Patients at high risk	0.80–1.21	2.40–3.63	7.99–12.09	0.84–1.50	2.51–4.49	8.36–14.96	0.88–1.74	2.63–5.23	8.76–17.42
Preexposure long-term prophylaxis									
Entire population	0.04–0.11	0.13–0.34	0.42–1.13	0.07–0.19	0.21–0.58	0.71–1.92	0.10–0.28	0.30–0.83	1.00–2.76
Patients at high risk	0.04–0.10	0.11–0.30	0.38–1.02	0.07–0.18	0.20–0.55	0.67–1.85	0.10–0.28	0.30–0.83	0.99–2.77
Postexposure short-term prophylaxis	0.18–1.14	0.54–3.42	1.82–11.40	0.30–1.94	0.91–5.81	3.04–19.37	0.43–2.78	1.30–8.35	4.32–27.83

*Base-case estimates refer to the point estimates detailed in Table A1. Minimum and maximum estimates refer to applying minimum and maximum values of health-related outcome measures detailed in Table A1.
